# Alkaline Ni-Zn Microbattery Based on 3D Hierarchical Porous Ni Microcathode with High-Rate Performance

**DOI:** 10.3390/mi14050927

**Published:** 2023-04-25

**Authors:** Gongchuan You, Zhe Zhu, Yixue Duan, Linfeng Lv, Xiaoqiao Liao, Xin He, Kai Yang, Ruiqi Song, Yi Yang, Liang He

**Affiliations:** 1 School of Mechanical Engineering, Sichuan University, Chengdu 610065, China; 2 State Key Laboratory of Advanced Technology for Materials Synthesis and Processing, Wuhan University of Technology, Wuhan 430070, China; 3 Department of Orthopedics, Orthopedic Research Institute, West China Hospital, Sichuan University, Chengdu 610041, China; 4 Med+X Center for Manufacturing, West China Hospital, Sichuan University, Chengdu 610041, China

**Keywords:** Ni-Zn microbattery, hierarchical porous structure, high rate, large capacity

## Abstract

Miniaturized energy storage devices with superior performance and compatibility with facile fabrication are highly desired in smart microelectronics. Typical fabrication techniques are generally based on powder printing or active material deposition, which restrict the reaction rate due to the limited optimization of electron transport. Herein, we proposed a new strategy for the construction of high-rate Ni-Zn microbatteries based on a 3D hierarchical porous nickel (Ni) microcathode. With sufficient reaction sites from the hierarchical porous structure as well as excellent electrical conductivity from the superficial Ni-based activated layer, this Ni-based microcathode is featured with fast-reaction capability. By virtue of facile electrochemical treatment, the fabricated microcathode realized an excellent rate performance (over 90% capacity retention when the current density increased from 1 to 20 mA cm^−2^). Furthermore, the assembled Ni-Zn microbattery achieved a rate current of up to 40 mA cm^−2^ with a capacity retention of 76.9%. Additionally, the high reactivity of the Ni-Zn microbattery is also durable in 2000 cycles. This 3D hierarchical porous Ni microcathode, as well as the activation strategy, provides a facile route for the construction of microcathodes and enriches high-performance output units for integrated microelectronics.

## 1. Introduction

With the rapid development of electronics towards multi-functions, ultra miniaturization and rational hetero integration, the miniaturized electrochemical energy storage devices with high performance are increasingly in demand [1,2,3,4]. During the past decade, microbatteries (MBs) and microsupercapacitors (MSCs) were witnessed as the promising micro-power sources in this field [5,6]. Unfortunately, an inferior energy supply severely restricts the practical applications of MSCs, and the applicability of MBs is also affected by the critical issues of safety, compatibility and insufficient power performance [7,8]. For typically developed MBs, some designs such as Swiss roll microelectrodes have been proposed with high energy on a small footprint area [9,10,11]. Furthermore, aqueous zinc (Zn)-based batteries, especially the alkaline ones, are regarded as competitive alternatives due to their high safety, enhanced voltage plateau and abundant resource [12,13,14]. However, the most reported alkaline MBs still suffer from unsatisfactory electrochemical performance in energy/power densities and cycling stability, which obstructs the pervasive applications [15,16].

Typically, as a highly developed reaction system associated with proton insertion and extraction, the alkaline reaction mechanism and structural advantages for high energy/power densities and electrochemical stability were thoroughly studied. Many reports proposed the cathode with a nanostructure design and various compositions to realize the improved capacity supply, reaction rate and electrochemical stability of nickel (Ni)-Zn batteries, such as the Ni@NiO core–shell structure [17], nickel–cobalt double hydroxides [18], etc. However, all the achievements are based on powder techniques, requiring optimal electron transport and ion diffusion [19,20]. For microelectrodes constructed via some microfabrication techniques such as deposition and printing [21,22], high areal energy density always requires a thick microelectrode to be constructed. When more active materials are loaded at a given footprint, the reactivity will always decrease due to the accompanied higher reaction impedance and longer diffusion distance, resulting in larger polarization and an even worse rate performance [23,24]. To ease the circumstance, some studies follow conventional strategies such as defect design engineering or the addition of a carbon nanotube to maintain mediocre reactivity. For example, Ma and coworkers [25] prepared a multilayered, printed microelectrode with effective ion and electron-transfer pathways, enabling a robust rate capability for a thick microelectrode. Recently, there were some reports about the concept of electroconverted Ni metal in energy storage, which could deliver ultrahigh reactivity in the assembled MBs [26]. This approach was originally studied in the field of catalysis, which rearranged atoms of atomic layers for the formation of epitaxial new phases in the two-dimensional structure of the surface layer [27,28]. The resulting new phases are always induced via repeated redox activations in an alkaline electrochemical environment. By utilizing this strategy in micro/nano structural Ni with hydroxides product, a highly promising microcathode with excellent electrical conductivity and highly reactive sites could be attained. Nevertheless, the capacity supply is poor on the premise of high-rate performance due to the insufficient specific surface of a Ni structure as well as its shadow depth. Regarding this, it is challenging to construct Ni-Zn MB with both high reactivity and large capacity.

We previously reported nanoporous Ni as the electroconversion target for energy storage, thus achieving durable Ni-Zn MB with ultrahigh-rate capability [26]. However, the prepared nano-structural nickel was very thin, and the provided capacity was far from satisfactory. Herein, we proposed electroconverted three-dimensional (3D) hierarchical porous nickel as the microcathode for construction of the high-rate Ni-Zn MB. Compared with a pure nanostructure, a 3D hierarchical porous skeleton is larger and more porous, enabling an enhanced reaction surface. Meanwhile, both the preparation of a metallic structure and the activation method are based on electrochemical treatment, which is facile and compatible with a miniaturized conductive substrate for the final rechargeable microelectrode. Additionally, the electroconversion process is generally about the surface transformation, and superior electron transport for the subsequent charge/discharge reaction is obtained. Consequently, both a high-rate performance and large capacity are ensured for the activated microcathode based on 3D hierarchical porous nickel. The reaction capacity of 0.159 mAh cm^−2^ is realized, and the rate density can be amplified to 20 mA cm^−2^ with a remarkable capacity retention of 90.49%. In the final assembled Ni-Zn MB, a capacity of 0.268 mAh cm^−2^ is obtained due to this reactivation in a highly concentrated alkaline environment, and the charge/discharge current can be expanded to 40 mA cm^−2^. Meanwhile, this excellent fast-charging performance can stably operate over 2000 cycles.

## 2. Materials and Methods

### 2.1. Preparation of an Activated 3D Hierarchical Porous Ni Microcathode

The preparation of 3D hierarchical porous nickel was based on the template of a hydrogen bubble. By applying a very negative bias, there were severe hydrogen evolution reactions accompanied by the electrodeposition of nickel, hence creating micropores for the nickel skeleton. In the preparation process, we utilized a CHI 760E electrochemical workstation (Shanghai CH Instruments Co., Shanghai, China) for the electrochemical operation. A typical three-electrode system was employed, in which the customized miniaturized nickel plate (effective area: 0.2 cm × 0.5 cm), saturated calomel electrode and Pt plate were served as the working electrode, reference electrode and counter electrode, respectively. Setting a cathodic potential of 6 V in the solution of 2 M NaCl, 2 M NH_4_Cl and 0.1 M NiCl_2_, the deposition of 3D hierarchical porous nickel can be completed in several minutes. After washing the electrodeposited microelectrode with deionized water a few times, an alkaline solution of 1 M KOH was prepared for the electroconversion process. Herein, a three-electrode system was also required, consisting of an electrodeposited microelectrode, Hg/HgO electrode and Pt plate as the working electrode, reference electrode and counter electrode, respectively. Through utilizing the procedure of cyclic voltammetry (CV) with the potential range of 0.2–0.6 V at a scan rate of 10 mV s^−1^, the electroconversion was saturated within 750 cycles.

Safety tip: During the electrodeposition of the 3D hierarchical porous nickel, both the chlorine and hydrogen will be released. Additionally, this preparation process is suggested to be conducted in a fume hood.

### 2.2. Preparation of Zinc Microanode

We also conducted the preparation of a zinc microelectrode via the electrochemical treatment as well. The similar, customized, miniaturized nickel plate was served as the working electrode, and the ordinary zinc plate worked as the counter electrode. By applying a constant voltage of −1.5 V for 30 min in a solution of 6 M KOH dissolved with 0.3 M ZnO, an electrodeposited zinc microanode was prepared.

### 2.3. Assembly of Ni-Zn MB

After the preparation of microcathode and microanode, the gel alkaline electrolyte was applied to assemble the Ni-Zn MB. Firstly, we slowly added 500 mg of sodium polyacrylate into a 20 mL solution of 6 M KOH dissolved with 0.3 M ZnO [29]. After stirring for 30 min, the transparent gel electrolyte was formed. Then, we selected two films tailored from a plastic bag as the package materials. After sealing three edges of the two films by a sealer machine, the activated 3D hierarchical porous nickel microcathode and electrodeposited zinc microanode were put inside the micro pocket and separated by two thin cardboard. Finally, we transferred the gel electrolyte into the MB and removed the separatecardboard, therefore the packaged Ni-Zn MB was obtained after the final edge was sealed.

### 2.4. Characterizations of Structure and Composition

The morphologies, microstructural and component characteristics of the samples were measured using a field-emission scanning electron microscope (FE-SEM, ZEISS Gemini 300) and transmission electron microscope (TEM, F200X) with an acceleration voltage of 200 kV. The chemical states and atomic structure information were investigated by X-ray photoelectron spectroscopy (XPS, Thermo Scientific K-Alpha) and Raman spectroscopy (WiTech alpha300R, with a 532 nm excitation from an argon ion laser).

### 2.5. Electrochemical Measurements

The electrochemical measurements about the activated Ni microcathode and Ni-Zn MB were performed on a CHI760E via procedures of CV and galvanostatic charge/discharge (GCD) tests. In detail, the single microelectrode was tested in a three-electrode alkaline system (Hg/HgO electrode as the reference electrode in 1 M KOH electrolyte) and the Ni-Zn MB was packaged (gel electrolyte of 6 M KOH with saturated ZnO) for the test. Additionally, the cycling test of the Ni-Zn MB was conducted on a Land cycler (Wuhan Kingnuo Electronic Co., Wuhan, China). The specific capacity (*C*) of the single microelectrode or assembled MB was calculated by the formulas *C* = *It*/*A*, where *I* was the discharge current density (mA cm^−2^) and *A* was the effective area of the microelectrode or the packaged MB (cm^2^).

## 3. Results and Discussion

**Figure 1:**
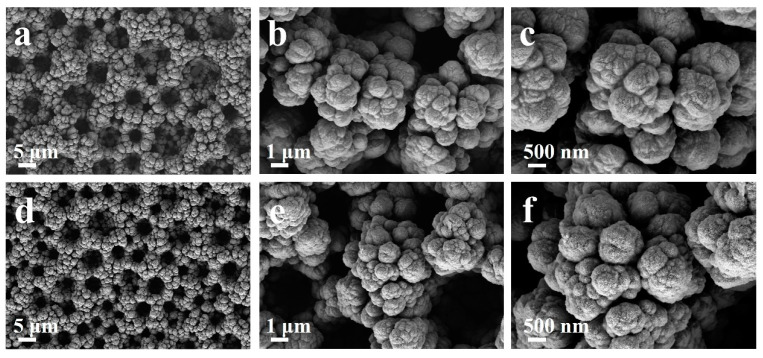
SEM images of (**a**–**c**) the original 3D hierarchical porous nickel and (**d**–**f**) the activated nickel structure for comparison, showing a rougher surface with small spikes.

The morphologies and structures of the original 3D hierarchical porous nickel and the activated one are demonstrated in Figure 1. It can be clearly observed that the pores are uniformly distributed in the Ni skeleton with a size of ~5 μm (Figure 1a). Furthermore, because they were affected by the hydrogen bubbles, it can be seen that the nickel structures are not smoothly connected. There are nanopores in the connection part, constituting the hierarchical porous structure (Figure 1b,c). When the activation treatment was applied, there was not much change in the major morphology (Figure 1d) and the surface morphology turned rougher with some small spikes, which can be observed at a higher magnification in Figure 1e,f. We can speculate that the activation process roughened the metallic surface when the composition transformation occurred.

**Figure 2:**
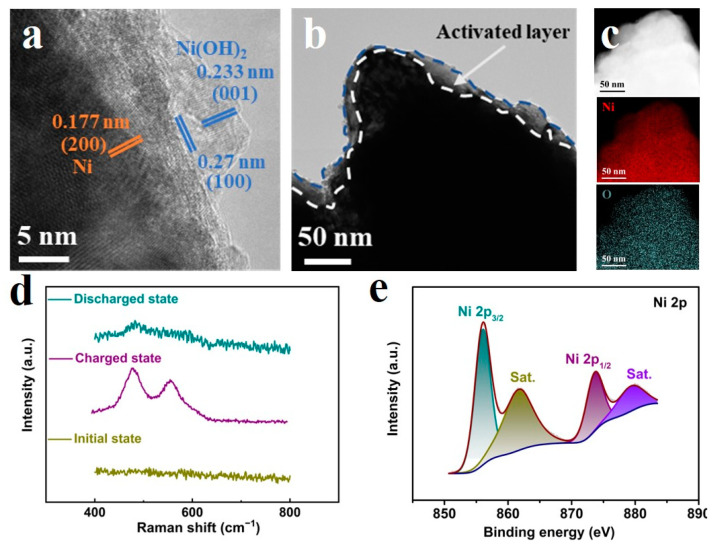
(**a**) HRTEM image, (**b**) TEM image and (**c**) the element mapping images of activated hierarchical porous nickel. (**d**) Raman spectra of samples at different states during the activation process and (**e**) XPS Ni 2p signals of the final activated Ni microelectrode.

To further analyze the composition of the activated layer, TEM and high-resolution TEM (HRTEM) images were recorded. As can be observed in Figure 2a, the *d* spacings regarding the activated lattice fringes were 0.233 nm and 0.27 nm, corresponding to (001) and (100) crystal planes of Ni(OH)_2_ (JCPDS No. 001-1047), thus revealing that the deposited metallic nickel was partly activated. For the inner layer, the *d* spacing of 0.177 nm clearly referred to the (200) crystal plane of Ni (JCPDS No. 001-1258). This magnified area is shown to be exactly the activated layer in Figure 2b, thus indicating that the activated layer (~10 nm) was maintained at the surface, and the conductivity of such an activated electrode was excellent (Figure S1). In Figure 2c, the energy-dispersive spectroscopy (EDS) mappings reflected a uniform distribution of Ni and O elements, thus supporting that the activation was almost uniform for the whole surface. This phase transformation was also characterized via Raman spectroscopy and XPS. As shown in Figure 2d, we measured the hierarchical porous Ni microelectrode at different states in a single activation cycle. When this Ni structure was charged from the initial state, there were typical γ-NiOOH signals with *E_g_* (477 cm^−1^) and *A_1g_* (557 cm^−1^) peaks [30,31]. Meanwhile, when the oxidized state was discharged back, a weak band around 486 cm^−1^ suggested a transformation from NiOOH to α-Ni(OH)_2_/NiO [32]. Furthermore, the final discharged state was also proven by the XPS result. In Figure 2e, the signals of 856.02 and 873.6 eV can be clearly identified as Ni 2p peaks for Ni(OH)_2_ [33,34]. All the results confirmed the hydroxide product after the activation.

**Figure 3:**
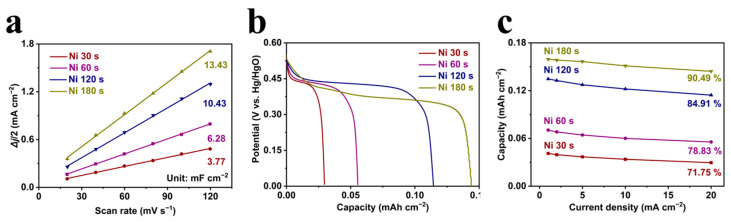
(**a**) The capacitive current versus scan rate, (**b**) discharge curves at the current density of 20 mA cm^−2^ and (**c**) the rate performance of the microelectrodes with different deposition time.

The electrochemical performance was associated with the excellent reaction transport, we prepared four microelectrodes with different deposition time for evaluation. As shown in Figure 3a, the electrochemically active surface area (ECSA) of different loadings was represented by the electrochemical double layer capacitance (*C*_dl_), measured by the fitted slope between the average current at 0.05 V from a series of CV tests with the potential ranging from 0 to 0.1 V versus Hg/HgO and its related scan rates (Figure S2) [35]. The *C*_dl_ of different microelectrodes with Ni deposition time of 30 s, 60 s, 120 s and 180 s were calculated to be 3.77, 6.28, 10.43 and 13.43 mF cm^−2^, respectively. Following the typical reaction between Ni(OH)_2_ and NiOOH (Figure S3), the gradually increased ECSAs well presented a load gradient for the reactivity analysis. In Figure 3b, the discharge capacities of various microelectrodes are calculated to be 0.029, 0.055, 0.114 and 0.143 mAh cm^−2^, which correspond to the increased loading. Notably, even though this activated metal electrode was featured with excellent electrical conductivity, the expanded impedance from increased active materials also slightly influenced the reaction polarization. Even at a high current density of 20 mA cm^−2^, the voltage plateau of a microelectrode with a deposition time of 180 s exhibited a minor decrease when compared with three other microelectrodes. Furthermore, the rate performance was utilized to evaluate the reactivity of the activation strategy when more loading/capacity was applied for a given microelectrode. As clearly observed in Figure 3c, the microelectrodes with increased capacity from more loading exhibit a higher rate performance. When the current density increased from 1 to 20 mA cm^−2^, the capacity retention of the activated microelectrodes with deposition time of 30 s, 60 s, 120 s and 180 s were calculated to be 71.75%, 78.83%, 84.91% and 90.49%, respectively. This improved performance could be ascribed to the increased reaction sites when a more metallic structure was loaded. Additionally, the excellent electron transport environment greatly eased the deteriorating effect from the increased impedance when more loading was applied, guaranteeing the fast-reaction capability for enhanced capacity.

**Figure 4:**
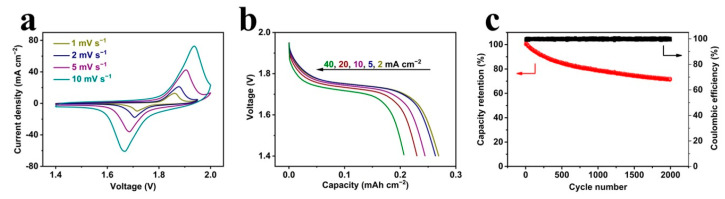
(**a**) CV curves at various scan rates, (**b**) discharge curves at various current densities and (**c**) the cycling performance of the assembled Ni-Zn MB.

We finally demonstrated the practical performance of this activated microcathode in assembled Ni-Zn MB with an electrodeposited Zn microanode (Figures S4–S6). The electrochemical performance was evaluated by CV curves, galvanostatic discharge profiles and a cycling test. It can be observed that there was no obvious change in the CV shape when the scan rate was varied from 1 to 10 mV s^−1^, supporting good electrochemical reversibility (Figure 4a). The related redox peaks can be ascribed to the following electrochemical reaction: Zn + 2NiOOH + 2KOH + 2H_2_O ⇌ K_2_[Zn(OH)_4_] + 2Ni(OH)_2_ [36]. Meanwhile, it should be noted that the electrolyte in full MB was changed to 6 M KOH with the addition of ZnO to ensure the matched fast reactivity in the microanode. In this process, a reactivation reaction occurred to the previously prepared microcathode, and the final stable capacity was enhanced. In the discharge curves of the assembled Ni-Zn MB at various current densities (Figure 4b), the rate current was expanded to 40 mA cm^−2^, and the discharge capacities were 0.268, 0.263, 0.244, 0.23 and 0.206 mAh cm^−2^ at the current density of 2, 5, 10, 20 and 40 mA cm^−2^, respectively. To further test the stability of fast charge/discharge, the cycling performance of the Ni-Zn MB was investigated at 40 mA cm^−2^. As shown in Figure 4c, the assembled MB delivered a stable cycling performance with a capacity retention of 71.23% in 2000 cycles along with nearly 100% Coulombic efficiency. Compared with the currently developed alkaline MBs (Table S1), all these performances clearly indicate the successful assembly of the Ni-Zn MB, the high reactivity brought by the activated metallic structure with an increased loading, and MBs with durable fast charging for practical applications in microelectronics.

## 4. Conclusions

In summary, a high-rate Ni-Zn microbattery with a large capacity was realized through the electroconversion approach of 3D hierarchical porous nickel. By virtue of the bubble template, the uniform micropores around the major metallic skeleton as well as the nanopores around the nickel connections constituted the whole hierarchical porous structure. Driven by the repeated redox activation, the surface activation slightly roughened the nickel surface and enabled superior electrical conductivity. Consequently, this unique, activated microcathode delivered an excellent rate performance; such high reactivity can withstand the increase in capacity loading with a slightly deteriorated performance. The prepared, activated nickel microcathode delivered a capacity of 0.159 mAh cm^−2^ and an excellent rate performance (over 90% capacity retention when the current density increased from 1 to 20 mA cm^−2^). The final assembled Ni-Zn MB achieved a large capacity of up to 0.268 mAh cm^−2^, an expanded rate current density of 40 mA cm^−2^ and an excellent cycling stability with over 70% retention after 2000 fast-charging cycles. This unique metallic construction, as well as the activation approach, provides an effective strategy for developing large-capacity MBs with high-rate performance.

## Supplementary Materials

The following supporting information can be downloaded at: https://www.mdpi.com/article/10.3390/mi14050927/s1, Figure S1: Photograph of direct conductivity test of reconstructed 3D hierarchical porous nickel microelectrode; Figure S2: CV curves of (a) 30 s, (b) 60 s, (c) 120 s, and (d) 180 s microelectrodes with the potential ranging from 0 to 0.1 V versus Hg/HgO at various scan rates; Figure S3: The first charge/discharge curve of Ni 30 s microelectrode at a current density of 5 mA cm^−2^; Figure S4: SEM images of electrodeposited Zn; Figure S5: The first charge/discharge curve of Zn microelectrode at a current density of 5 mA cm^−2^; Figure S6: Photograph of the assembled Ni-Zn microbattery; Table S1: Comparison of electrochemical performance with recently reported MBs and other state-of-the-art micro energy-storage systems (the rate performance here refers to the current range and the related capacity retention (%)).
