# Supplementary material for: Alkaline Ni-Zn Microbattery Based on 3D Hierarchical Porous Ni Microcathode with High-Rate Performance
Source: Micromachines (Basel). 2023 Apr 25;14(5):927. doi: 10.3390/mi14050927 (PMC10224541; doi:10.3390/mi14050927)
Supplement: Supplementary file 1 [file micromachines-14-00927-s001.zip › micromachines-2302662-supplementary.pdf]

Alkaline Ni-Zn Microbattery Based on 3D Hierarchical Porous Ni Microcathode with High-Rate Performance

Gongchuan You [1,†], Zhe Zhu [1,†], Yixue Duan [1,2], Linfeng Lv [1,2], Xiaoqiao Liao [1], Xin He [1], Kai Yang [1], Ruiqi Song [1], Yi Yang [3,*] and Liang He [1,2,4,*]

1 School of Mechanical Engineering, Sichuan University, Chengdu 610065, China
2 State Key Laboratory of Advanced Technology for Materials Synthesis and Processing, Wuhan University of Technology, Wuhan 430070, China
3 Department of Orthopedics, Orthopedic Research Institute, West China Hospital, Sichuan University, Chengdu 610041, China
4 Med+X Center for Manufacturing, West China Hospital, Sichuan University, Chengdu 610041, China
* Correspondence: hxyangyi@163.com (Y.Y.); hel20@scu.edu.cn (L.H.)
† These authors contributed equally to this work.

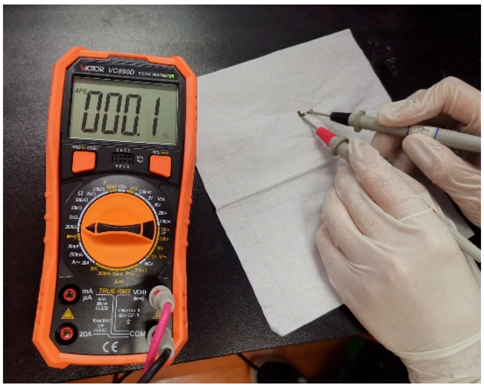

Figure S1. Photograph of direct conductivity test of reconstructed 3D hierarchical porous nickel microelectrode.

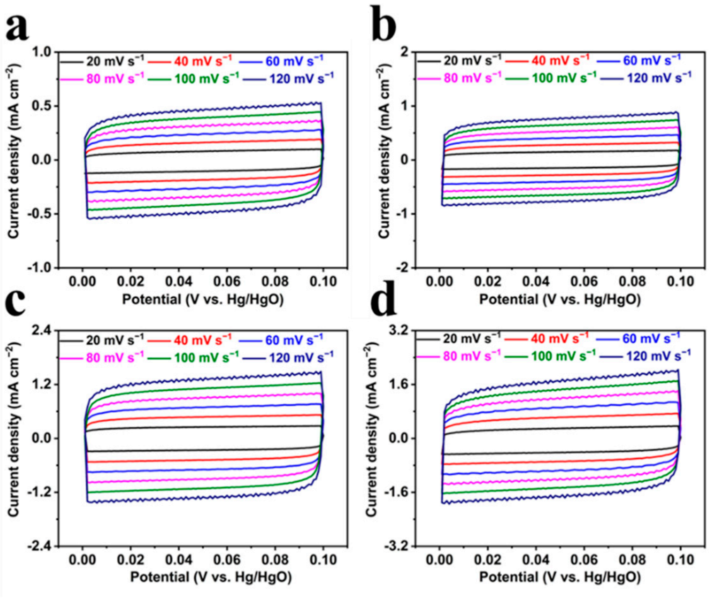

Figure S2. CV curves of (a) 30 s, (b) 60 s, (c) 120 s, and (d) 180 s microelectrodes with the potential ranging from 0 to 0.1 V versus Hg/HgO at various scan rates.

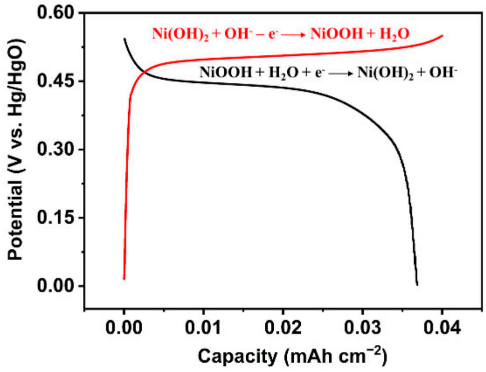

Figure S3. The first charge/discharge curve of Ni 30 s microelectrode at a current density of 5 mA cm^{-2}.

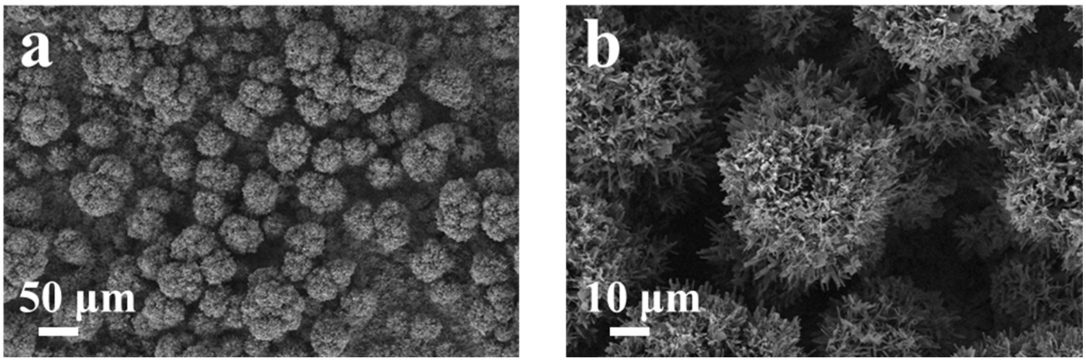

Figure S4. SEM images of electrodeposited Zn.

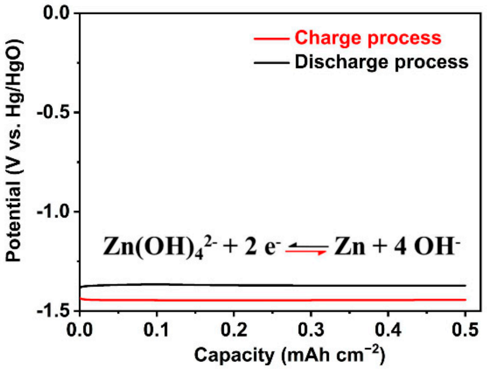

Figure S5. The first charge/discharge curve of Zn microelectrode at a current density of 5 mA cm^{-2}.

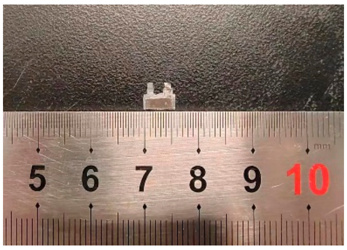

Figure S6. Photograph of the assembled Ni-Zn microbattery.

(Notice: The gel electrolyte should be prepared in a thin and almost solid state, otherwise the assembled MB is easy to be short or the packed area needs to be extended.)

Table S1. Comparison of electrochemical performance with recently reported MBs and other state-of-the-art micro energy-storage systems (the rate performance here refers to the current range and the related capacity retention (%)).

Cathode // Anode materials	Device type	Capacity (mAh cm^{-2})	Rate performance	Cycling stability (%)	Ref.
Reconstructed porous Ni // Zn	Alkaline MB	0.268	2 to 40 mA cm^{-2} 76.8 %	71.2 in 2000 cycles	This work
Ni-Co LDH@CC // Zn		0.108	0.5 to 1 mA cm^{-2}	91 in 1000 cycles	1
$Co(OH)_2$@NiCo LDH // Zn		0.108	1 to 10 mA cm^{-2} 39.8 %	71 in 800 cycles	2
Ni@$Ni(OH)_2$ // Zn		0.152	1 to 30 mA cm^{-2} 59.8 %	74.6 in 1800 cycles	3
Ni-$Ni(OH)_2$/$Zn(OH)_2$ // Zn		0.149	1 to 200 mA cm^{-2}, 85.9 %	91.2 in 3500 cycles	4
Ag // Zn		0.167	0.4 to 4 μA cm^{-2} 46.3 %	84.2 in 100 cycles	5

References

1. Tian, Z.; Sun, Z.; Shao, Y.; Gao, L.; Huang, R.; Shao, Y.; Kaner, R. B.; Sun, J. Ultrafast rechargeable Zn micro-batteries endowing a wearable solar charging system with high overall efficiency. *Energy Environ. Sci.* **2021**, *14*, 1602-1611.
2. Wang, Y.; Hong, X.; Guo, Y.; Zhao, Y.; Liao, X.; Liu, X.; Li, Q.; He, L.; Mai, L. Wearable Textile-Based Co-Zn Alkaline Microbattery with High Energy Density and Excellent Reliability. *Small* **2020,** *16*, 2000293.
3. Hao, Z.; Xu, L.; Liu, Q.; Yang, W.; Liao, X.; Meng, J.; Hong, X.; He, L.; Mai, L., On-Chip Ni-Zn Microbattery Based on Hierarchical Ordered Porous Ni@$Ni(OH)_2$ Microelectrode with Ultrafast Ion and Electron Transport Kinetics. *Adv. Funct. Mater.* **2019,** *29*, 1808470.
4. Zhu, Z.; Kan, R.; Wu, P.; Ma, Y.; Wang, Z.; Yu, R.; Liao, X.; Wu, J.; He, L.; Hu, S.; Mai, L. A Durable Ni-Zn Microbattery with Ultrahigh-Rate Capability Enabled by In Situ Reconstructed Nanoporous Nickel with Epitaxial Phase. *Small* **2021,** *17,* 2103136.
5. Bi, S.; Wan, F.; Wang, S.; Jia, S.; Tian, J.; Niu, Z. Flexible and tailorable quasi-solid-state rechargeable Ag/Zn microbatteries with high performance. *Carbon Energy* **2021,** *3*, 167–175.
